# An mRNA Profiling Study of Vaginal Swabs from Pre- and Postmenopausal Women

**DOI:** 10.3390/cimb45080411

**Published:** 2023-08-07

**Authors:** Elena Chierto, Federica Alessandrini, Carla Bini, Eugenia Carnevali, Matteo Fabbri, Paolo Fattorini, Pierangela Grignani, Francesca Scarnicci, Pamela Tozzo, Andrea Verzeletti, Susi Pelotti, Loredana Buscemi, Carlo Robino

**Affiliations:** 1 Department of Public Health Sciences and Pediatrics, University of Turin, 10126 Turin, Italy; carlo.robino@unito.it; 2 Section of Legal Medicine, Department of Biomedical Sciences and Public Health, Polytechnic University of Marche, 60126 Ancona, Italy; 3 Department of Medical and Surgical Sciences, Unit of Legal Medicine, University of Bologna, 40126 Bologna, Italy; 4 Unità Operativa Semplice Laboratory of Forensic Science, Section of Legal Medicine, Department of Medicine and Surgery, S. Maria Hospital, University of Perugia, 05100 Terni, Italy; 5 Section of Legal Medicine, Department of Translational Medicine, University of Ferrara, 44121 Ferrara, Italy; 6 Department of Medicine, Surgery and Health, University of Trieste, 34129 Trieste, Italy; 7 Section of Legal Medicine and Forensic Sciences, Department of Public Health, Experimental and Forensic Medicine, University of Pavia, 27100 Pavia, Italy; 8 Section of Legal Medicine, Department of Health Surveillance and Bioethics, Università Cattolica del Sacro Cuore, 00168 Rome, Italy; 9 Department of Molecular Medicine, University of Padua, 35121 Padua, Italy; 10 Department of Medical and Surgical Specialties, Radiological Sciences and Public Health, Forensic Medicine Unit, University of Brescia, 25123 Brescia, Italy; 11 AOU Ospedali Riuniti Ancona, Polytechnic University of Marche, 60126 Ancona, Italy

**Keywords:** vaginal mucosa, mRNA profiling, body fluid identification, CYP2B7P1, MUC4, MYOZ1

## Abstract

Body fluid identification by means of mRNA profiling provides valuable supplementary information in forensic investigations. In particular, the detection of vaginal mucosa mRNA markers is highly relevant in sexual assault cases. Although the vagina undergoes characteristic age-related physiological changes over a lifetime, few studies have evaluated the efficacy of vaginal mRNA markers in women of different ages. In this multicentric study, a 19-plex mRNA profiling assay including vaginal-specific markers (CYP2B7P1, MUC4, MYOZ1) was tested in a collection of 6–20-month-old vaginal swabs obtained from pre- (*n* = 84) and postmenopausal (*n* = 55) female volunteer donors. Overall, participating laboratories were able to correctly identify ~85% of samples as vaginal mucosa by mRNA profiling. The assay’s success rate did not differ between the two age groups and was not affected by the time interval between swab collection and RNA analysis. MYOZ1 resulted a less sensitive vaginal marker compared to MUC4 and CYP2B7P1. A significant relative increase in the contribution to the total amplification signal was observed for MUC4, compared to CYP2B7P1 and MYOZ1, in postmenopausal women. Observation of other body fluids and tissues different from vaginal mucosa was also evaluated in connection to information on previous sexual activity and menstrual cycle phase at the time of sampling.

## 1. Introduction

In order to identify the donor of transferred biological traces deposited at the crime scene or on a person, DNA typing is a routine practice worldwide. However, a DNA profile does not reveal the circumstances by which it was transferred. Since each cell type is characterized by a unique pattern of gene expression, highly specific RNA biomarkers can be used to identify the tissue source of the DNA found [1]. For this reason, in the past few years, mRNA profiling based on reverse transcriptase (RT) PCR followed by end-point multiplex PCR and detection of amplified targets by capillary electrophoresis has emerged as a powerful tool for the identification of body fluids of forensic interest [2].

Given the possibility of simultaneously isolating both RNA and DNA from the same forensic sample [3], mRNA profiling can be easily combined with DNA genotyping. In order to be functional, mRNA profiling assays need to target a wide range of different body fluids and tissues of potential forensic interest, such as venous blood, saliva, nasal mucosa, semen, vaginal mucosa, menstrual secretion, and skin [4,5,6]. In particular, the ability to identify vaginal epithelia or their secretions as the source of a DNA profile can significantly aid the investigation of sexual assault cases. In the case of sexual assault, in fact, there may be a transfer of material from the aggressor to the victim, but also vice versa [7]. The proof of the vaginal origin of the material found on the body or on the clothes of the suspect is therefore an essential element for the reconstruction of the dynamics of the crime. This is especially true when the cognitive abilities of the offended person, for example, young children or disabled people, do not allow direct acquisition of the testimonial data, or they can be more easily subjected to external manipulation.

It is well known from the scientific literature that the vaginal mucosa is an extremely changeable environment, whose characteristics change as a result of multiple hormonal factors related to age (prepubertal period, fertile phase, post menopause), and possible concurrence of physiological (pregnancy) or pathological processes of an infectious or dystrophic/dysplastic type [8]. It is therefore reasonable to believe that the expression of some of the vaginal mRNAs currently proposed for forensics may be influenced, in whole or in part, by these factors. However, almost all the experimental studies conducted up to now to identify and evaluate candidate vaginal mRNA have been based on a small number of donors [5,9,10,11,12,13,14]. Even when larger samples were investigated, they were poorly differentiated by age and pathophysiological characteristics of female donors [15,16].

With this in mind, the Italian working group of the International Society for Forensic Genetics (Genetisti Forensi Italiani, GeFI) promoted among the partner laboratories the collection of a large sample of vaginal swabs from female volunteers, belonging to two different age groups (fertile age and post menopause), in order to perform an interlaboratory study for the preliminary validation of the mRNA profiling method specifically focusing on the detection of three specific markers for the vaginal mucosa [17]. The present study aims to expand the analysis published in [17], integrating the results with those subsequently received from further GeFI laboratories, for a total of ten participating groups.

The present study focused on a set of proposed forensic vaginal mRNA markers (CYP2B7P1, MUC4, MYOZ1), part of the 19-plex mRNA profiling assay described by [4].

*CYP2B7P1* (cytochromeP450, family 2, subfamily B, polypeptide 7 pseudogene 1) is a pseudogene related to the cytochrome P450 gene family [18]. *MUC4* (mucin 4) encodes for an integral membrane glycoprotein and is expressed in the endocervix where it protects epithelial surfaces of the reproductive tract against pathogens and controls sperm entry into the uterus [19,20,21]. *MYOZ1* (myozenin-1) is a member of the myozenin family. Members of this gene family function as calcineurin-interacting proteins that help tether calcineurin to the sarcomere of cardiac and skeletal muscle [22]. Although the function of the two gene transcripts CYP2B7P1 and MYOZ1 in vaginal mucosa is unknown, they appear to be highly specific markers for this tissue [11].

While primarily aimed at evaluating the effectiveness of MUC4, CYPB7P1, and MYOZ1 as mRNA profiling vaginal markers in pre- (PR-M) and postmenopausal (PO-M) women, the study also allowed to address other forensically relevant aspects of mRNA profiling. These included the impact on the quality of obtained results of time elapsed between sample collection and laboratory analysis, and of the different instrumentation and analytical settings adopted by participating laboratories. The expression of other body fluids and tissues different from vaginal mucosa included in the mRNA profiling panel (blood, menstrual secretions, seminal fluid, saliva, nasal mucosa, and skin) was also investigated in connection with available information on previous sexual activity and phase of the menstrual cycle at the time of specimen collection from donors.

## 2. Materials and Methods

### 2.1. Sample Collection

The tested vaginal swabs were collected through self-sampling by adult consenting PR-M (*n* = 84) and PO-M (*n* = 55) women. The mean age was 34.4 ± 8.5 SD in the PR-M subsample and 58.0 ± 7.7 SD in the PO-M subsample. Samples were anonymized in order to prevent a link to the original donors, who were also asked to provide information regarding the menstrual cycle phase at the time of sampling and sexual activity in the 10 days before sampling. After collection, vaginal swabs were dried and stored at room temperature for a variable period of time (6–20 months). Sets of swabs (*n* = 13–14), homogeneous for the age category and mean time since swab collection, were prepared (see Table S1 for details). Blinded sample sets were then distributed to the ten GeFI participating laboratories, which had been previously involved in a GeFI preparatory exercise, organized in collaboration with the Netherlands Forensic Institute, aimed at building competency in DNA/RNA co-analysis and mRNA profiling [23].

### 2.2. DNA/RNA Extraction, Quantitation, and Reverse Transcription

DNA/RNA co-extraction (AllPrep DNA/RNA Micro or Mini kit, Qiagen, Hilden, Germany), DNase treatment (TURBO DNA-free™ Kit, Invitrogen, Carlsbad, CA, USA), and reverse transcription (SuperScript™ IV First-Strand Synthesis System, Invitrogen, Carlsbad, CA, USA) were performed as described in [24]. Quantitation experiments of total human and human male DNA isolated from vaginal swabs were all performed in a single laboratory using the Plexor^®^ HY System (Promega, Madison, WI, USA) and CFX96 Touch Real-Time PCR detection system (Bio-Rad Laboratories, Hercules, CA, USA).

### 2.3. Amplification and Detection of mRNA Markers

mRNA profiling was performed using a 19-plex mRNA assay that, beside vaginal mucosa markers, MUC4, CYP2B7P1, and MYOZ1, targets: blood (ALAS2: 5′-Aminolevulinate Synthase 2, CD93: Complement component C1q receptor, HBB: Hemoglobin Subunit Beta), saliva (HTN3: Histatin 3, STATH: Statherin), nasal mucosa (BPIFA1: BPI Fold Containing Family A Member 1, STATH: Statherin), seminal fluid (SEMG1: Semenogelin 1, KLK3: Kallikrein Related Peptidase 3), spermatozoa (PRM1: Protamine 1), menstrual secretions (MMP7: Matrix metalloproteinase 7, MMP10: Matrix metalloproteinase 10, MMP11: Matrix metalloproteinase 11), skin (CDSN: Corneodesmosin, LCE1C: Late Cornified Envelope 1C), and two reference housekeeping genes (ACTB: Actin Beta, 18S-rRNA: 18S ribosomal RNA) [4]. Initially, each sample was amplified using three different cDNA inputs: 0.2, 1, and 3.75 µL. mRNA profiling results were detected by capillary electrophoresis (CE). Each participant laboratory used the available CE instrumentation and settings previously defined through internal laboratory validation (see Table S2 for details). Optimal cDNA input identified through inspection of electropherograms was then used in PCR replicates with a total of four PCR replicates for each vaginal sample.

### 2.4. Scoring Method of mRNA Profiling Results

Scoring of mRNA profiling results was conducted according to [25]. In brief, a specific body fluid/tissue was considered as “observed” in a sample if “x ≥ n/2”, and “not observed” if 0 ≤ x < n/2, where “n” corresponds to the maximum number of specific electrophoretic peaks for a given body fluid/tissue which can be observed overall in the four replicates, and “x” corresponds to the number of specific peaks for a given body fluid/tissue actually observed in the four replicates. For example, vaginal mucosa was observed in a sample if, in the four replicates (*n* = 12), there was a total of at least 6 peaks above the analytical threshold in correspondence with the vaginal markers CYP2B7P1, MUC4, and MYOZ1.

### 2.5. Statistical Analysis

Unpaired t-tests were calculated for two data set comparisons when data followed a normal distribution, otherwise the Mann–Whitney test was performed. The Chi-square test was employed to compare frequencies. One-way analysis of variance (ANOVA) with Bonferroni post hoc test was used for comparison among multiple groups when data followed a normal distribution, otherwise the Kruskal–Wallis with Dunn’s post hoc test was performed. To test for normality, the d’Agostino–Pearson test was used. The Spearman rank test was used in order to assess the relationship between two variables. Data are expressed as mean values of n experiments ± SE. All statistical analyses were performed with the software GraphPad Prism 6.0 (GraphPad Software, San Diego, CA, USA).

## 3. Results

### 3.1. mRNA Profiling Success Rate

Optimal cDNA input values adopted by each participating laboratory are listed in Table S2. It can be seen that, for the majority of the swabs (63.3%), an optimal cDNA input of ≤0.2 µL was identified. Among samples requiring optimal cDNA inputs ≥1 µL, 76.5% were processed in three laboratories, all adopting an ABI Prism 310 Genetic Analyzer.

**Figure 1:**
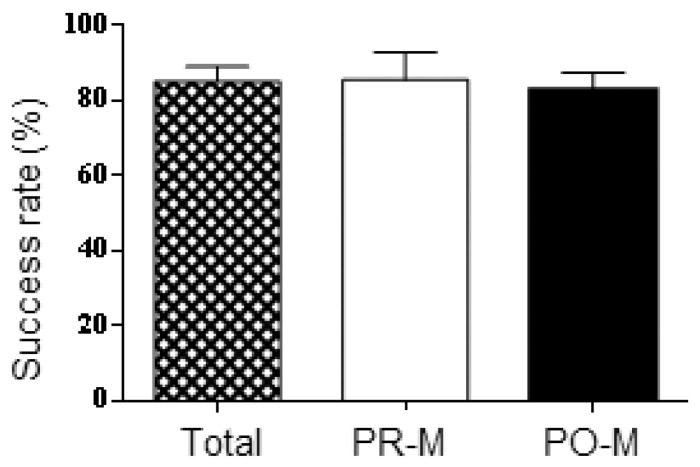
mRNA profiling success rates (vaginal mucosa observed, according to the scoring method) across laboratories in the whole sample (Total) and within subcategories of PR-M and PO-M women. Data are expressed as mean ± SE.

Despite the wide range of cDNA inputs adopted by participants, no significant difference in mRNA profiling success rates was observed across laboratories considering whole sample sets provided to each laboratory (Figure 1). On average, vaginal mucosa was observed according to scoring guidelines in 85% (±4% SE) of the samples. The same could be said for the average mRNA profiling success rates within each age category: 86% (±7% SE) for PR-M samples, and 83% (±5% SE) for PO-M samples. Based on these results, the data obtained from the participating laboratories were considered cumulatively in the following analyses. Overall, vaginal mucosa was observed by mRNA profiling in 118 out of 139 total swabs (84.9%), 72 out of 84 PR-M swabs (85.6%), and 46 out of 55 PO-M swabs (83.6%).

**Figure 2:**
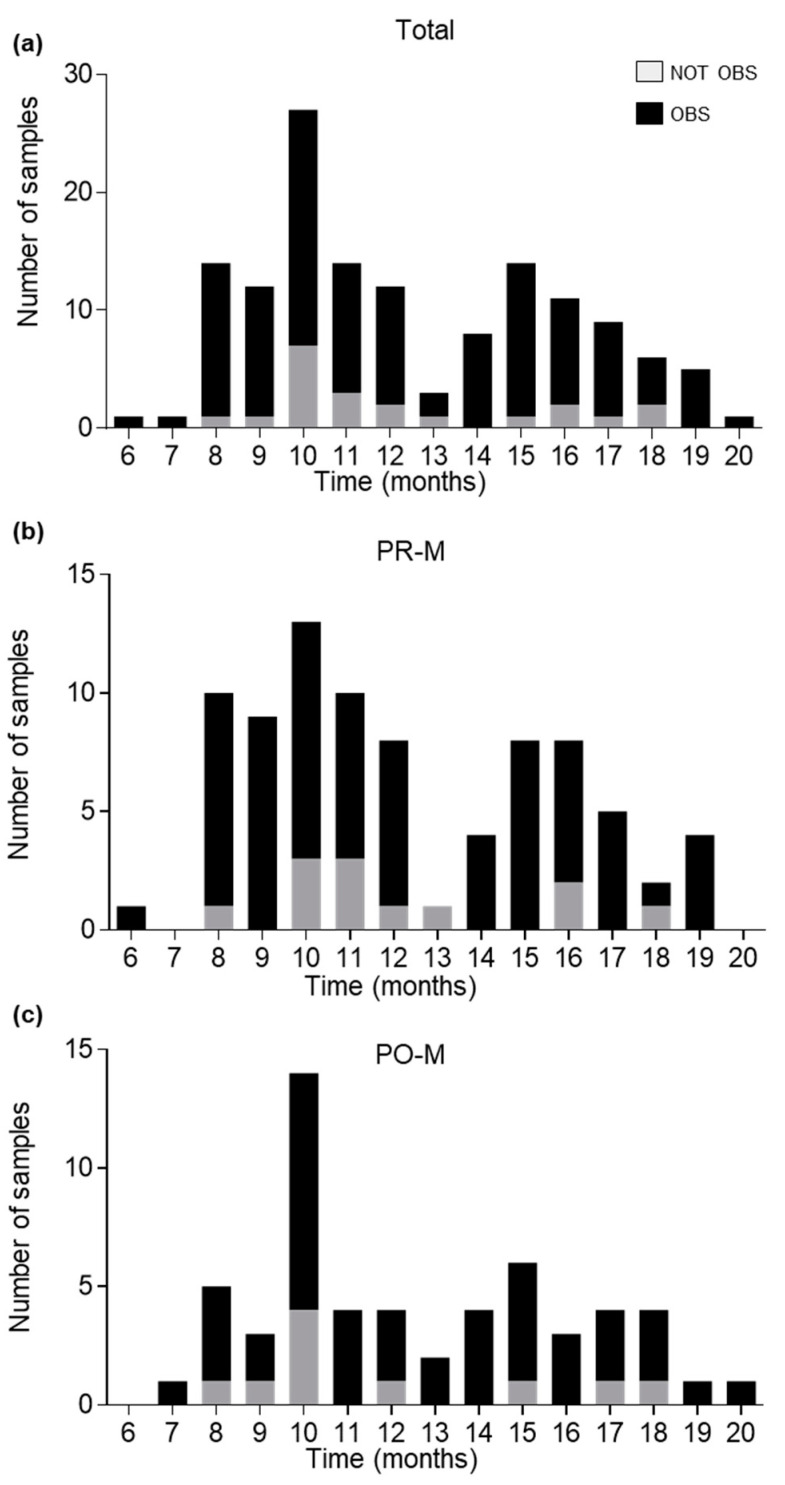
Distribution of tested samples according to time interval between swab collection and RNA extraction expressed in months (*x* axis) and mRNA profiling outcome considering the total number of samples (**a**), PR-M samples (**b**) and PO-M samples (**c**). Vaginal mucosa was “observed” (black) or “not observed” (gray), according to the scoring method.

No significant correlation was found between mRNA profiling success rate (vaginal mucosa “observed” according to scoring method) and the time interval between swab collection and mRNA analysis, which ranged from 6 to 20 months, with a median of 11 months in both the whole sample and the PR-M subsample, and of 12 months in the PO-M subsample (Figure 2).

### 3.2. Amplification Efficiency of Vaginal Markers

**Figure 3:**
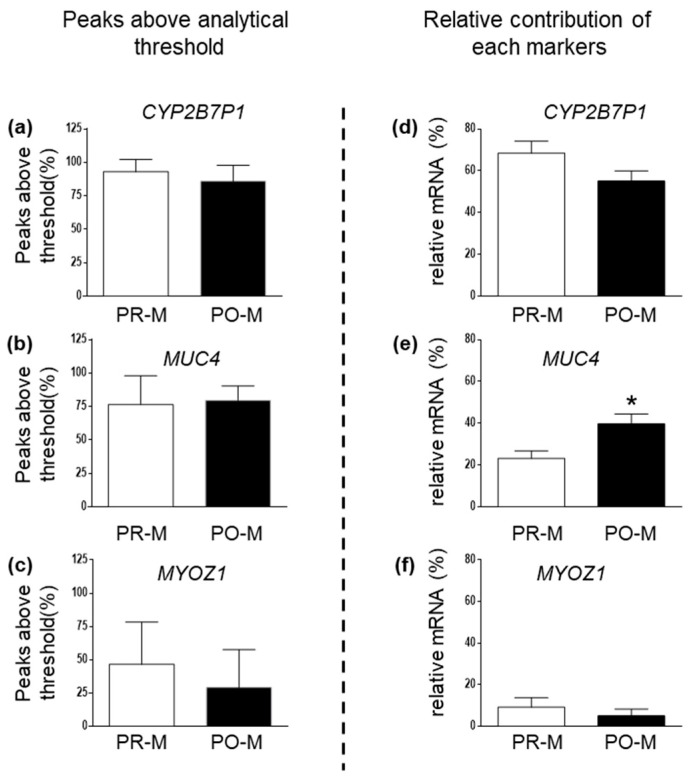
Percentage of peaks above the analytical threshold for the vaginal markers CYP2B7P1 (**a**), MUC4 (**b**), and MYOZ1 (**c**) in mRNA profiling replicates of PR-M (*n* = 84) and PO-M (*n* = 55) vaginal samples. Relative contribution of each gene to total peak height for the vaginal markers CYP2B7P1 (**d**), MUC4 (**e**), and MYOZ1 (**f**) in mRNA profiling replicates of PR-M (*n* = 75) and PO-M (*n* = 50) vaginal samples. Data are expressed as mean ± SE. *: *p* < 0.05 (*t*-test).

PCR efficiency of the three vaginal markers in mRNA profiling experiments conducted in the two different age groups is summarized in Figure 3. Pairwise differences in the percentage of peaks above the analytical threshold observed in mRNA profiling replicates from samples in the two age categories were not significant for any of the tested vaginal markers (Figure 3a–c). The relative contribution of CYP2B7P1, MUC4, and MYOZ1 to the cumulative peak height of vaginal signal (measured in rfu) observed in mRNA profiling replicates is shown in Figure 3d–f. In this case, data from one participating laboratory that reported several off-scale peaks for CYP2B7P1 and MUC4 were not considered in calculations. A significant increase in average relative contribution to total peak height was observed for MUC4 in PO-M samples, compared to PR-M samples.

**Figure 4:**
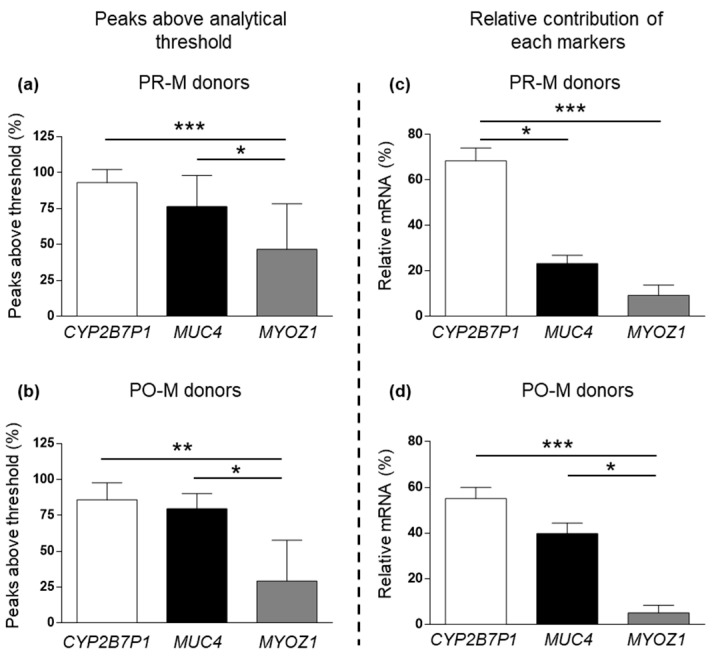
Percentage of peaks above the analytical threshold for the vaginal markers CYP2B7P1, MUC4, and MYOZ1 in PR-M (**a**) and PO-M vaginal samples (**b**). Relative contribution of each gene to total peak height for the vaginal markers CYP2B7P1, MUC4, and MYOZ1 in PR-M (**c**) and PO-M vaginal samples (**d**). Data are expressed as mean ± SE. *: *p* < 0.05; **: *p* < 0.01; ***: *p* < 0.001; (**a**) ANOVA with Tukey post hoc test; (**b**–**d**) Kruskal–Wallis with Dunn’s post hoc test).

Differential PCR efficiency between the three vaginal markers within mRNA profiling experiments is depicted in Figure 4. The percentage of peaks above the analytical threshold was significantly lower for MYOZ1 compared to MUC4 and CYP2B7P1, both in the PR-M and PO-M subsamples (Figure 4a,b). Similarly, the MYOZ1 average relative contribution to the cumulative peak height of all vaginal markers was significantly lower compared to CYP2B7P1 in both age categories (Figure 4c,d). In PR-M donors, CYP2B7P1 relative contribution was significantly higher compared to both MUC4 and MYOZ1, which otherwise did not significantly differ from each other (Figure 4c).

### 3.3. Other Body Fluids and Tissues in Vaginal Swabs

Body fluids and tissues other than vaginal mucosa observed by mRNA profiling in vaginal swabs are summarized in Table 1.

Skin was the most frequent additional tissue type, being observed in 41.7% of total vaginal samples. The occurrence of skin cross-reactivity was significantly higher in the PR-M subset (56%) compared to the PO-M subset (20%) (chi-square test, *p* < 0.001). Observation of skin was principally due to CDSN peaks above the analytical threshold, which were present in at least two of the four PCR replicates in 93% of the skin positive samples. A strong positive correlation (*p* < 0.001, Spearman rank test) was seen between skin observation and the amount of cDNA input in mRNA profiling experiments. Observation of skin was reported by nine out of ten laboratories, while sporadic observation of other tissue types such as nasal mucosa and/or saliva was limited to three laboratories (Figure S1).

In Table 2, observation of seminal fluid, spermatozoa, blood, and menstrual secretions are considered in connection with information on sexual activity (SA) in the 10 days before sampling (available for 138 donors) and menstruation at the time of sampling (available for 108 donors).

In donors who reported SA in the last 10 days (*n* = 59), seminal fluid and seminal fluid with spermatozoa were effectively observed only in 32.2% (*n* = 19) and 6.8% (*n* = 4) of the vaginal samples, respectively. The distribution across laboratories of donors who reported SA but no evidence of seminal fluid (with or without spermatozoa) in mRNA profiling results is rather homogeneous (Figure S2). Of these samples, 17.5% (*n* = 7) also displayed negative mRNA profiling results for vaginal mucosa, indicating that RNA degradation was likely. Notably, two out of seven of such samples still contained detectable male DNA according to total and male DNA quantitation experiments. DNA quantitation experiments of swabs from donors who reported SA in absence of observed seminal fluid (with or without spermatozoa) showed that 67.5% (*n* = 27) of them did not contain detectable male DNA, which was in agreement with negative mRNA profiling results.

Among donors who did not report SA (*n* = 79), seminal fluid (always without spermatozoa) was observed in 11.4% (*n* = 9) of the samples. DNA quantitation experiments confirmed that male DNA was not present in these samples. In contrast to what was observed for skin, no significant correlation was found between these unexpected observations of seminal fluid and the amount of cDNA input in mRNA profiling experiments. One single laboratory was responsible for 44.4% (*n* = 4) of such unexpected results (Figure S2).

In donors who reported menstruation (M) at the time of sampling (*n* = 11), menstrual secretion and blood were observed, in 36.4% (*n* = 4) and 45.5% (*n* = 5) of the swabs, respectively. One of these samples also returned negative mRNA profiling results to vaginal mucosa, suggesting RNA degradation. In donors who did not report M at the time of sampling including PO-M women (*n* = 97), menstrual secretion and blood were observed in 16.5% (*n* = 16) and 17.5% (*n* = 17) of the swabs, respectively. In particular, menstrual secretions were observed in 11.5% (*n* = 7) of PR-M donors (*n* = 61) and 25.0% (*n* = 9) of PO-M donors (*n* = 36). The distribution across laboratories of observations of blood and/or menstrual secretion related to reported M is displayed in Figure S3. It could be seen that, whereas mRNA profiling negative results in M donors were rather homogenously distributed, 50% (*n* = 8) of the observations of blood and/or menstrual secretion in non-M donors (*n* = 25) came from a single laboratory. No significant correlation was seen between the observation of menstrual secretion and/or blood according to the scoring method and the amount of cDNA input in mRNA profiling experiments.

## 4. Discussion

The obtained results confirmed that the combination of MUC4, CYP2B7P1, and MYOZ1 is a reliable indicator of vaginal mucosa, with ~85% of vaginal swabs correctly identified by mRNA profiling. The apparently reduced success rate observed here, compared to previous interlaboratory studies focusing on the same set of markers [12], can be explained by the more stringent criteria leading to conclusions regarding the presence of vaginal mucosa in the tested samples, which were based on replication of PCR results rather than the observation of marker-specific peaks in single amplification experiments. Also confirmed was the prolonged stability of mRNA in vaginal samples [26], with tested swabs successfully analyzed after up to 20 months of storage at room temperature.

In general, the identification of vaginal mucosa was equally successful in pre- and postmenopausal donors (~86% and ~84%, respectively). When considering the informativity of each proposed vaginal marker, it must be noted that MUC4 was originally selected for forensic mRNA profiling through a combination of literature and database searches [21], and it was already known to be expressed both in pre- and postmenopausal women [27]. On the contrary, CYP2B7P1 and MYOZ1 were identified through transcriptome profiling (RNA-Seq) of vaginal swab samples obtained from two donors, aged 26 and 30 years old [11]. The obtained results confirm the expression of MUC4 in both age categories. However, a significant relative increase in MUC4 contribution to the total peak height of vaginal markers, at the expense of CYP2B7P1 and MYOZ1, was seen in post- compared to premenopausal donors. Although these results were obtained by end-point PCR and will therefore need further verification through quantitative PCR gene expression analysis, they could possibly reflect physiological age-related changes in the vaginal transcriptome. It would therefore be advisable in the future to extend the evaluation of candidate vaginal mRNA markers to donors of different age categories, including prepubescent girls, in order to better assess their suitability in the forensic molecular investigation of child abuse cases. Studies of this kind are presently almost absent in the forensic literature. Negative results for vaginal mucosa mRNA markers included in the 19-plex used in this study have been sporadically reported in young girls [12], highlighting the need for a more detailed investigation. End-point PCR mRNA profiling experiments also indicated the absence of significant differences in the expression of CYP2B7P1 and MYOZ1 between the two age categories. In the tested 19-plex mRNA profiling assay, the amplification efficiency of MYOZ1 was confirmed to be constantly reduced compared to MUC4 and CYP2B7P1 [23], irrespective of the age category of donors.

When considering other body fluids detected in vaginal swabs by mRNA profiling, discrepancies were found between observed and expected results according to previous sexual activity and menstrual cycle phase at the time of swab collection as reported by donors. In the first case, it must be remembered that while donors were asked to report sexual activity up to 10 days before swab collection, it is known that the persistence of seminal traces still being detectable by molecular (DNA) methods in the vagina rarely exceeds 6 days [28]. Accordingly, no observation of seminal fluid and/or spermatozoa was seen by mRNA profiling in donors reporting sexual activity whose swabs resulted negative for male DNA in quantitation experiments. If just considering swabs from donors who reported sexual activity in which vaginal mucosa was successfully observed, thus excluding the possibility of mRNA degradation, samples negative for seminal mRNA markers but that contained male DNA were 22%. A possible explanation of mRNA profiling results that appeared discordant with sexual activity information obtained from donors may simply be that tissue-specific marker detection is mainly influenced by the initial amount of target biological fluid [29]. Therefore, failure of mRNA profiling results is expected to affect primarily other body fluids possibly present in low amounts in the vagina, such as seminal fluid, rather than overabundant vaginal transcripts. As a justification for discordant results, it must also be stressed that donors’ anonymous reports accompanying the vaginal swabs did not differentiate between protected and unprotected sexual activity, and did not include information regarding ejaculation. Another class of discordant results was the observation of seminal fluid in mRNA profiling experiments conducted on swab extracts from donors who did not report sexual activity and did not display male DNA in their DNA counterpart (~11%). Cross-reactivity of seminal mRNA markers with vaginal mucosa appears to be a rare event [30]. While preferential detection of seminal markers in mixed stains subjected to mRNA profiling has been shown [31], genuine cross-reactivity appears unlikely. Since prevention measures were adopted to detect carryover and contamination (RT-minus and end-point PCR negative controls), the uneven distribution of semen discordant results across laboratories suggests that cDNA overloading coupled with inadequate preliminary setting of CE conditions may have led to spurious amplification products above the analytical threshold. Notably, the single laboratory that reported the largest ratio of discordant results for seminal fluid used a recently introduced CE instrument (SeqStudio), different from the one (ABI PRISM 310) they adopted in the mRNA profiling exercise preparatory to this study [23]. The obtained results thus highlight the importance of performing extended validation experiments, before the implementation of the 19-plex mRNA profiling assay on newly adopted CE platforms.

Menstrual secretion and blood markers were frequently not observed (~55–65%) in donors who reported menstruation at the time of sampling. This result can be also explained by the stringent criteria chosen to interpret mRNA profiling results, which required ≥50% of the tissue-specific peaks to be present in replicates. Menstrual secretions were often observed, according to the adopted interpretation criteria, in donors who did not report menstruation at the time of sampling (~16%). Genuine cross-reactivity cannot be excluded in such cases. In particular, possible expression throughout the menstrual cycle of MMP7, MMP10, and MMP11 have been previously described [5,9,12]. Observation of menstrual secretions in postmenopausal donors (25%) also highlights the need for further expression studies on menstrual markers MMP7, MMP10, and MMP11 in this age category. A similar rate of positive results (~18%) was also observed for blood in donors who did not report menstruation at the time of sampling. Blood-specific markers can be sporadically detected in vaginal swabs [5,32]. However, for the same reasons previously outlined to explain seminal fluid discordant results in mRNA profiling, the prevailing occurrence of blood observations (without menstrual secretions) in a limited number of participating laboratories points to cDNA overloading and suboptimal choice of CE conditions as a possible cause of such results.

While cDNA overloading could also partly explain the common observation of skin in vaginal swabs [4], genuine cross-reactivity appears the most relevant factor in this case, given the high rate of observations across laboratories, including those that never reported “unexpected” results for semen/blood. Frequent cross-reactivity of skin markers, in particular CDSN, with vaginal mucosa is indeed well described [32]. A recent collaborative study on mRNA profiling conducted by FoRNAP (Forensic RNA Profiling) group also demonstrated that skin markers are very sensitive and they tend to show up in other body fluids [33]. Notably, the observation of skin was significantly higher in premenopausal donors. The two skin markers included in the 19-plex mRNA profiling assay, CDSN and LCE1C, both encode for proteins related to stratum corneum structure and functionality [34,35]. It is known that the vaginal epithelium, after thickening and developing a distinct cornified layer during the reproductive years, thins following menopause with its stratum corneum displaying variable degrees of keratinization [36]. The observed difference can therefore possibly reflect age-related modifications of transcription patterns in vaginal mucosa.

In conclusion, the present study confirms that mRNA profiling is a promising molecular tool for the reconstruction of sexual crime dynamics, enabling body fluid identification in up to 20-month-old vaginal samples from adult females.

On the other hand, there are evident limitations participants must address before routine implementation of the proposed forensic mRNA assay is possible. In several laboratories, observation of “unexpected” body fluids in vaginal samples was frequent, indicating that careful preliminary optimization of cDNA input is necessary in order to avoid nonspecific results due to tissue cross-reactivity. Moreover, definition of analysis parameters, such as analytical threshold, which may also contribute to reduce nonspecific observations, should be customized and updated for different models of capillary electrophoresis platforms. Further inter- and intra-laboratory validation exercises are therefore needed to refine mRNA proofing workflow and to develop suitable frameworks for the reporting of results.

## Figures and Tables

**Table 1:** Observation rates of body fluids/tissues other than vaginal mucosa, according to the scoring method. Inclusion in the mRNA profiling assay of marker PRM1 specific for spermatozoa allowed to discriminate between seminal fluid and seminal fluid with spermatozoa.

	Observed %	Not Observed %
Skin	41.7	58.3
Saliva	5.8	94.2
Nasal mucosa	3.6	96.4
Seminal fluid	20.1	79.9
Seminal fluid + spermatozoa	2.9	97.1
Blood	20.9	79.1
Menstrual secretions	19.4	80.6

**Table 2:** (a) Observation of seminal fluid with and without spermatozoa (spz) in vaginal swabs from donors who provided information on sexual activity (SA) in the 10 days before sampling; (b) Observation of menstrual secretion and blood in vaginal swabs from donors with and without menstruation (M) at the time of sampling. (“OBS” means observed according to the scoring method; “NOT OBS” means not observed according to the scoring method).

(a)	SA (*n* = 59)		NO SA (*n* = 79)	
	OBS	NOT OBS	OBS	NOT OBS
Seminal fluid	32.2	67.8	11.4	88.6
Seminal fluid + spz	6.8	93.2	0.0	100.0
(b)	M (*n*= 11)		NO M (*n* = 97)	
	OBS	NOT OBS	OBS	NOT OBS
Menstrual secretion	36.4	63.6	16.5	83.5
Blood	45.5	54.5	17.5	82.5

## Data Availability

The data that support the findings of this study are available from the corresponding author upon reasonable request.

## Supplementary Materials

The following supporting information can be downloaded at: https://www.mdpi.com/article/10.3390/cimb45080411/s1, Figure S1: Observation of saliva, nasal mucosa, and skin in vaginal samples across laboratories; Figure S2: Distribution across laboratories of observations of seminal fluid considering the information about sexual activity reported by vaginal swab donors; Figure S3: Distribution across laboratories of observations of blood and/or menstrual secretion considering the information about the menstrual cycle reported by vaginal swab donors; Table S1: Characteristics of vaginal swabs subsets assigned to each participating laboratory; Table S2: Capillary electrophoresis platforms and analysis settings adopted by participating laboratories.
