# Supplementary material for: An mRNA Profiling Study of Vaginal Swabs from Pre- and Postmenopausal Women
Source: Curr Issues Mol Biol. 2023 Aug 7;45(8):6526–37. doi: 10.3390/cimb45080411 (PMC10453267; doi:10.3390/cimb45080411)
Supplement: Supplementary file 1 [file cimb-45-00411-s001.zip › Figure_S2.pdf]

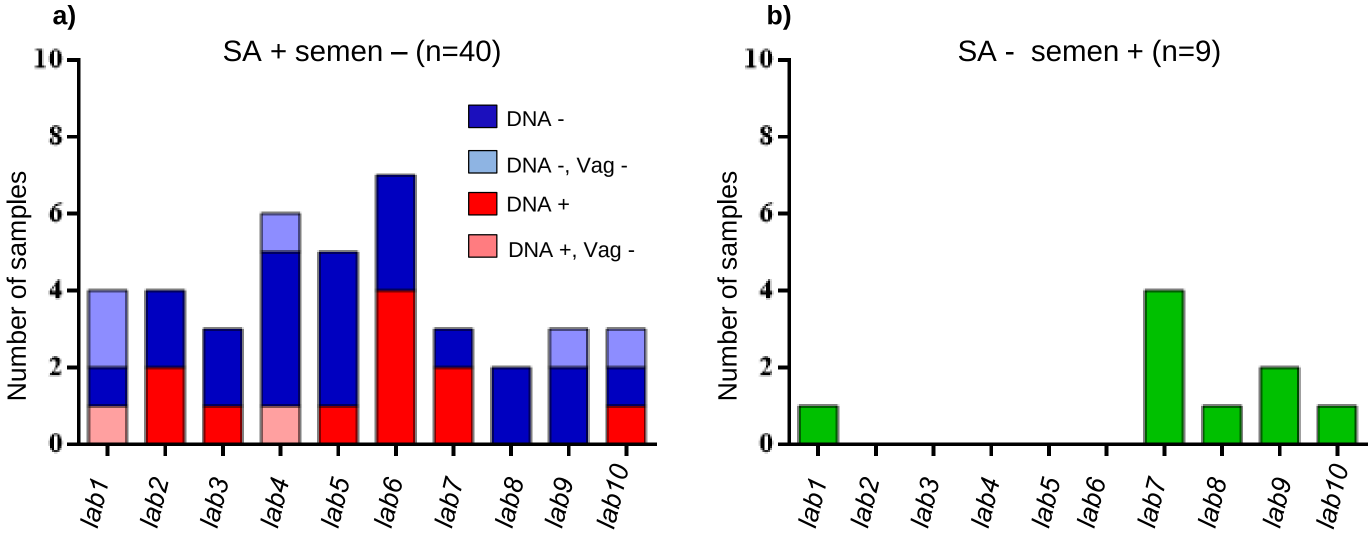

Figure S2: Distribution across laboratories of observations of seminal fluid considering the information about sexual activity reported by vaginal swab donors a) Negative mRNA profiling results for seminal fluid and/or spermatozoa in donors who reported sexual activity (SA +). DNA+: male DNA detected in DNA quantitation experiments. DNA-: male DNA not detected in DNA quantitation experiments. Vag -: vaginal mucosa not observed by mRNA profiling. b) Observations of seminal fluid in donors who did not report sexual activity (SA -).
