# Supplementary material for: An mRNA Profiling Study of Vaginal Swabs from Pre- and Postmenopausal Women
Source: Curr Issues Mol Biol. 2023 Aug 7;45(8):6526–37. doi: 10.3390/cimb45080411 (PMC10453267; doi:10.3390/cimb45080411)
Supplement: Supplementary file 1 [file cimb-45-00411-s001.zip › Figure_S3.pdf]

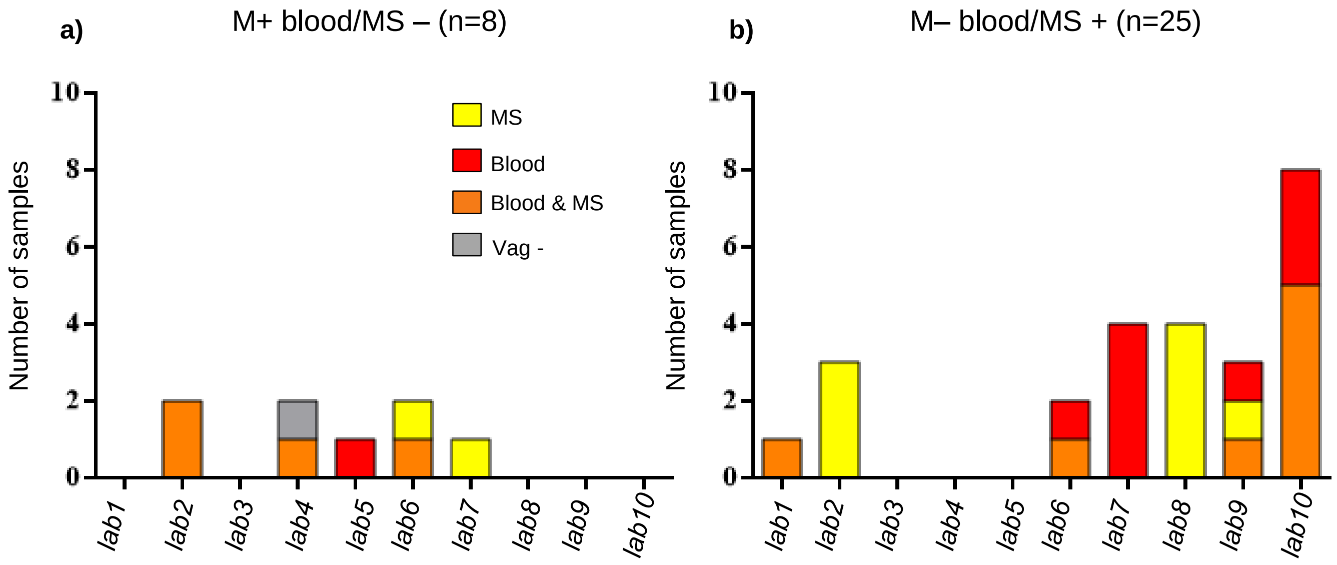

Figure S3:Distribution across laboratories of observations of blood and/or menstrual secretion considering the information about the menstrual cycle reported by vaginal swab donors a) Negative mRNA profiling results for blood and/or menstrual secretion (MS) in M donors (M+). Vag - : vaginal mucosa not observed by mRNA profiling. b) Observations of blood and/or MS in non M donors (M-).
