# Supplementary material for: An mRNA Profiling Study of Vaginal Swabs from Pre- and Postmenopausal Women
Source: Curr Issues Mol Biol. 2023 Aug 7;45(8):6526–37. doi: 10.3390/cimb45080411 (PMC10453267; doi:10.3390/cimb45080411)
Supplement: Supplementary file 1 [file cimb-45-00411-s001.zip › Table_S1_REV.pdf]

	PR-M swabs	PO-M swabs	Time in months between swab collection and RNA extraction (mean ± SD)
**lab 1**	8	6	11,7 ± 2,8
**lab 2**	9	5	12,7 ± 3,0
**lab 3**	8	6	11,7 ± 3,7
**lab 4**	8	6	12,7 ± 3,1
**lab 5**	9	5	13,2 ± 3,7
**lab 6**	8	6	12,0 ± 3,8
**lab 7**	8	5	12,5 ± 4,0
**lab 8**	8	6	12,5 ± 3,2
**lab 9**	9	5	11,0 ± 3,8
**lab 10**	9	5	13,7 ± 3,2

Table S1: Characteristics of vaginal swabs subsets assigned to each participating laboratory. Swabs were collected from pre- (PR-M) and postmenopausal (PO-M) women.
