# Supplementary material for: An mRNA Profiling Study of Vaginal Swabs from Pre- and Postmenopausal Women
Source: Curr Issues Mol Biol. 2023 Aug 7;45(8):6526–37. doi: 10.3390/cimb45080411 (PMC10453267; doi:10.3390/cimb45080411)
Supplement: Supplementary file 1 [file cimb-45-00411-s001.zip › Table_S2_REV.pdf]

	Genetic Analyzer	Polymer	Analytical threshold (rfu)	optimal cDNA input (ul)		
				**≤ 0.2**	**1**	**3.75**
**lab 1**	AB 3500	POP4	100	14	0	0
**lab 2**	ABI PRISM 310	POP4	100	0	9	5
**lab 3**	AB 3500	POP7	100	12	0	2
**lab 4**	ABI PRISM 310	POP4	75	14	0	0
**lab 5**	ABI PRISM 310	POP4	50	11	3	0
**lab 6**	ABI PRISM 3130	POP4	50	2	6	6
**lab 7**	SeqStudio	POP1	50	9	0	4
**lab 8**	ABI PRISM 310	POP4	150	14	0	0
**lab 9***	ABI PRISM 310	POP4	50	11(2)	2 (2)	1 (1)
**lab 10**	ABI PRISM 3130	POP4	50	1	6	7

Table S2: Capillary electrophoresis platforms and analysis settings adopted by participating laboratories. Optimal cDNA input amounts identified by laboratories are reported on the right.
**One laboratory (lab 9) used varying amounts of cDNA input in replicates of five samples. These samples, indicated by numbers within round brackets, were categorized according to the prevailing cDNA input used in the four replicates.*
