# Supplementary figures and images for: An mRNA Profiling Study of Vaginal Swabs from Pre- and Postmenopausal Women
Source: Curr Issues Mol Biol. 2023 Aug 7;45(8):6526–37. doi: 10.3390/cimb45080411 (PMC10453267; doi:10.3390/cimb45080411)

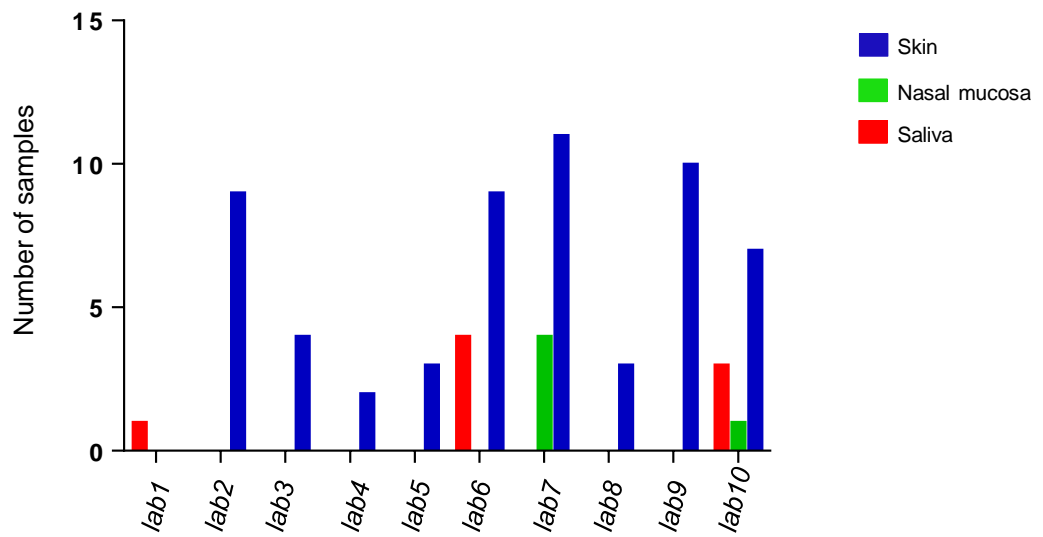

*Figure S1: Observation of saliva, nasal mucosa and skin in vaginal samples across laboratories.*

Supplement: Supplementary file 1 [file cimb-45-00411-s001.zip › Figure_S1.pdf]
